# Genome-wide analysis of chimpanzee genes with premature termination codons

**DOI:** 10.1186/1471-2164-10-56

**Published:** 2009-01-29

**Authors:** Anna Wetterbom, Ulf Gyllensten, Lucia Cavelier, Tomas F Bergström

**Affiliations:** 1 Department of Genetics and Pathology, Rudbeck Laboratory, Uppsala University, Uppsala, SE-751 85, Sweden; 2 Department of Animal breeding and Genetics, Swedish University of Agricultural Sciences, Uppsala, SE-750 07, Sweden

## Abstract

**Background:**

Premature termination codons (PTCs) cause mRNA degradation or a truncated protein and thereby contribute to the transcriptome and proteome divergence between species. Here we present the first genome-wide study of PTCs in the chimpanzee. By comparing the human and chimpanzee genome sequences we identify and characterize genes with PTCs, in order to understand the contribution of these mutations to the transcriptome diversity between the species.

**Results:**

We have studied a total of 13,487 human-chimpanzee gene pairs and found that ~8% were affected by PTCs in the chimpanzee. A majority (764/1,109) of PTCs were caused by insertions or deletions and the remaining part was caused by substitutions. The distribution of PTC genes varied between chromosomes, with Y having the highest proportion. Furthermore, the density of PTC genes varied on a megabasepair scale within chromosomes and we found the density to be correlated both with indel divergence and proximity to the telomere. Within genes, PTCs were more common close to the 5' and 3' ends of the amino acid sequence. Gene Ontology classification revealed that olfactory receptor genes were over represented among the PTC genes.

**Conclusion:**

Our results showed that the density of PTC genes fluctuated across the genome depending on the local genomic context. PTCs were preferentially located in the terminal parts of the transcript, which generally have a lower frequency of functional domains, indicating that selection was operating against PTCs at sites central to protein function. The enrichment of GO terms associated with olfaction suggests that PTCs may have influenced the difference in the repertoire of olfactory genes between humans and chimpanzees. In summary, 8% of the chimpanzee genes were affected by PTCs and this type of variation is likely to have an important effect on the transcript and proteomic divergence between humans and chimpanzees.

## Background

The genetic basis of the observed phenotypic divergence between humans and chimpanzees has since long intrigued scientists. Since humans and chimpanzees diverged 5–7 million years ago [1,2] their genomes have acquired a multitude of lineage specific mutations, including nucleotide substitutions, insertions and deletions (indels), duplications and inversions. The sequence divergence caused by substitutions and indels has been estimated to 1.2–1.5% [3-5] and 3–7% [3,4,6], respectively. Several hypotheses have been proposed to reconcile the observed overall low sequence divergence with the large phenotypic differences between humans and chimpanzees. These hypotheses include sequence divergence of protein coding genes [7], gain and loss of genes [8-11], differential gene expression [12-14] and divergent patterns of alternative splicing[15]. In this study we have focused on the role of nonsense mutations and frame shift indels that cause premature termination codons (PTCs) in chimpanzee protein coding genes. The dramatic effect of this type of mutations has been described in a number of human genes [16-18].

PTCs have the capability of changing the pattern of alternative splicing and expressed protein isoforms, since mRNAs affected by PTCs will either be degraded by nonsense-mediated decay (NMD) or translated into truncated protein sequences [19,20]. The coupling of PTCs and NMD has previously been suggested as a novel mechanism to regulate gene expression [21-24].

Although the occurrence of genes with premature stop codons in human genes has been described on a genome wide scale [25,26] there is no parallel study in the chimpanzee. Two previous studies [4,5] have examined PTCs in chimpanzee genes using the high quality genome sequence of chromosome 21 (previously referred to as chromosome 22 in the chimpanzee [27]). In the initial publication of the chimpanzee genome sequence the identification of PTCs was hampered by the low quality of the sequence [3]. By using the current (6×) chimpanzee assembly and stringent quality criteria we have overcome this problem and here we report the first genome wide study of PTCs in the chimpanzee. Chimpanzee specific PTCs were identified in 1,109 genes, which were further studied with respect to their genome location and context, biological function and the position of the PTC within genes.

## Results and discussion

### Detection of premature termination codons in chimpanzee protein-coding genes

**Figure 1:**
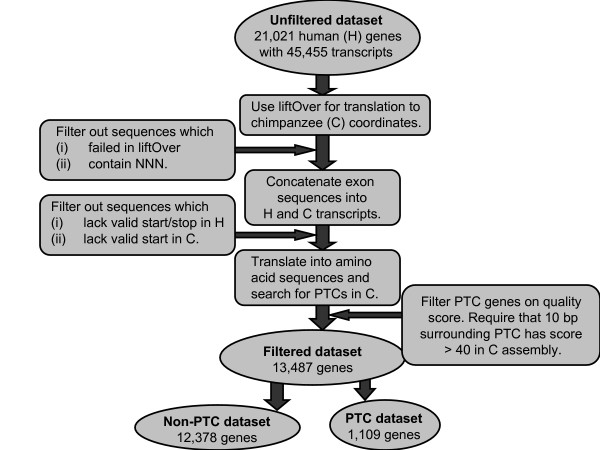
**Outline of the filtering process**. To obtain reliable predictions of PTCs in chimpanzee genes we applied a series of different filtering steps to remove genes where the PTCs were caused by unreliable gene annotations, lack of sequence or low quality of the chimpanzee assembly. A majority of the removed genes were filtered out because they failed to be correctly translated between the genomes (i.e. the liftOver step).

In an attempt to identify chimpanzee genes with PTCs we have analyzed pairs of human-chimpanzee genes. Annotations of all human protein coding genes were collected from the Ensemble database [28], resulting in a total of 21,021 genes and 45,455 associated transcripts. Chimpanzee genes and transcripts were inferred from human annotations and human exon coordinates were translated to the chimpanzee genome using the liftOver tool (available from the UCSC Genome Browser [28]). Translation between the genomes depends on a pairwise alignment (also available from the UCSC Genome Browser [28]) and we required all chimpanzee exons in a transcript to have the same orientation and be located on the same chromosome after the translation. Failure to translate coordinates accurately may be due to e.g. lack of sequence in a region or problems in aligning regions with large indels or lots of repeats. Both human and chimpanzee exon sequences were concatenated into complete coding sequences, translated into amino acid sequences and then screened for PTCs in the chimpanzee. Several filtering steps were employed to ensure that the detected PTCs were not a result of incorrect gene predictions or low quality of the chimpanzee assembly (see Figure 1 and the Methods section for details). Gene predictions were required to have correct start and stop codons in human and a correct start codon in chimpanzee. To account for the lower quality of the chimpanzee assembly we required the region surrounding a PTC to have a quality score > 40, corresponding to less then 1 sequencing error in 10,000 bp.

After filtering, a total of 13,487 genes and 26,779 associated transcripts remained. The genes were divided into two datasets: (i) PTC genes where one or several of the associated chimpanzee transcripts were affected by PTCs, and (ii) non-PTC genes where none of the annotated transcripts were affected by PTCs in the chimpanzee. In the present study all analyses will be based on genes instead of transcripts, thereby avoiding the bias introduced by different genes having different numbers of transcripts.

Approximately 8% (1,109 of 13,487) of the chimpanzee genes were affected by PTCs, indicating that the genes were either pseudo genes in the chimpanzee (in cases where all transcripts had PTCs) or that the genes had a different transcriptional pattern as compared to the corresponding human genes (in cases where the genes had both functional transcripts and transcripts with PTCs).

Furthermore, we determined the type of mutational event leading to the PTC. Indels were found to be the causative mutation in approximately 70% of the PTC genes (n = 764) and in the remaining 30% (n = 345) of the genes the PTC was caused by a substitution. The exact location of the premature termination codons and the type of mutational event is detailed in the Additional file 1 (Table S1).

### Characteristics of genes with PTCs in the chimpanzee

#### The density of PTC genes varies both between and within chromosomes

**Figure 2:**
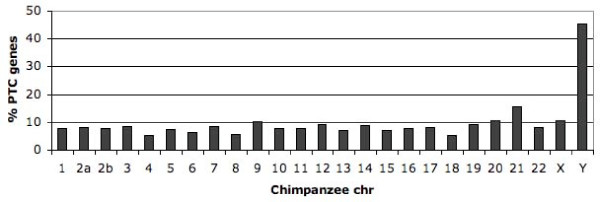
**Chromosomal distribution of PTC genes**. The x-axis denotes the chimpanzee chromosomes and the y-axis represents the % of genes that were affected by PTCs on a specific chromosome.

An estimated 8% of all chimpanzee genes were affected by PTCs. This figure is higher then the 5% we reported in a previous study of chromosome 21 [4] and the discrepancy is likely to be due to differences in density of PTC genes between chromosomes (Figure 2). In the present study, the proportion of PTC genes varied from 5 to 16% for the autosomes, and a significant difference between chromosomes was observed (Wilcoxon test, p < 6*10^-8^). The Y chromosome had a strikingly high percentage (> 40%) of PTC genes. The Y-chromosome is known to have an increased proportion of inactivating mutations in the chimpanzee lineage and a large number of pseudo genes [29,30], thereby explaining the increased fraction of PTC genes observed in this study. The proportion of PTC genes was next estimated for 1 Mbp-windows across all chromosomes and a significant variation between different chromosomal regions was observed (Wilcoxon test, p < 3*10^-16^). This suggests that the large-scale chromosomal structure influences the density of PTC genes, although the cause for this effect is unclear.

#### The proportion of PTC genes was correlated to both indel divergence and proximity to the telomere

Having noticed that the proportion of PTC genes varied both between chromosomes and between 1 Mb windows from the same chromosome, we sought to understand the underlying reasons. The density of PTC genes in a particular region may be affected by several genomic properties such as GC-content, presence of repeats, CpG-islands, segmental duplications, substitutions, indel divergence or the distance to the telomere or centromere. To study the relationship between the density of PTC genes and these genomic properties, we divided the chromosomes into non-overlapping 1 Mbp windows and applied a regression model, evaluating the relationship between the fraction of genes in the two datasets (PTC and non-PTC genes) and each genomic feature. Occurrence of PTC genes was found to be significantly associated with both indel divergence and distance to the telomere. The other genomic properties showed no correlation with the occurrence of PTC genes, or a similar correlation to non-PTC genes. The results indicate that PTC genes were in most respects similar to non-PTC genes.

The correlation with indel divergence (calculated as the % of bp located in indels) was higher for the PTC genes (Table 1) as compared to the non-PTC genes. The observed association was significant but rather weak, possibly as a result of the genomic alignment obtained from UCSC [28]. The indel divergence estimated from the alignment was only 0.64%, which was considerably lower than the 3–7% previously reported [3,4,6]. This is explained by the alignment parameters having been optimized not to allow for gaps longer then 100 bp. Although the majority of indels are shorter than 100 bp [3], longer indels contribute more to the indel divergence since they include more base pairs. Thus, given that indels > 100 bps were not taken into account, the correlation seen between PTC genes and indel divergence was most likely underestimated. A correlation between PTC genes and indel divergence was further supported by the fact that approximately 70% of the PTCs were actually caused by indel events.

**Table 1:** Summary of the regression analyses: predictor(s) of the density of PTC genes.

**Predictor(s) of PTC gene density**	**Adjusted R**	**p-value**
Distance to telomere	0.137	4*10^-15^
Indel divergence (% bp in indels)	0.087	2*10^-6^
Combined model (telomeric distance and indel divergence)	0.165	3*10^-16^

In addition to indel divergence, the proportion of PTC genes in a region increased with proximity to the telomere (Table 1). The closer to the telomere, the more likely the gene was to be affected by a PTC. Telomeres are known to have an increased indel divergence [3] and therefore we combined these two variables in a multiple regression model (Table 1). The combined model has a higher R-value then the previous regression analyses, suggesting that the two variables should be considered together.

#### PTCs were preferentially located in distal parts of the gene and in regions with less functional domains

**Figure 3:**
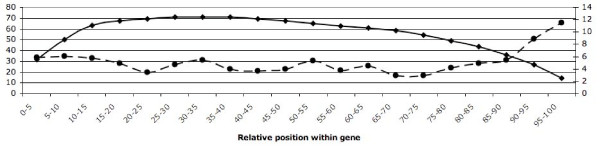
**Location of PTCs and Pfam matches within PTC genes**. The x-axis represents the relative position within the gene (0–5% of the length and so on), the left y-axis shows the % of all PTC genes with a Pfam match at a specific position (solid line and diamonds) and the right y-axis shows the % of all PTC genes with a PTC in the same interval (dashed line and dots).

To examine the relative location of PTCs within affected genes, each gene was partitioned into windows covering 5% of the length, and the proportion of genes with PTCs in a specific region was estimated. The results showed a higher frequency of PTCs in the distal parts of the genes than in the central region. When a PTC occurs early in the transcript, the nucleotide sequence will most likely not be translated into a protein but instead be subjected to NMD [19,20]. On the other hand, when the PTC occurs late in the transcript, translation it is likely to take place and result in a truncated protein sequence. To further investigate the consequences of PTCs we searched for a relationship between the location of PTCs and functional domains of the gene, using the Pfam database [31] to map functional domains onto the PTC genes. All PTC genes were matched against the Pfam database [31] and the relative location of Pfam hits was recorded. A negative correlation (*r *= -0.75, p-value < 0.0002) was found between the location of PTCs and the location of Pfam matches (Figure 3), suggesting a stronger selection against PTC in functional regions of genes. The results support the assumption that PTCs may be tolerable as long as the termination codon does not disrupt the structure of centrally located functional domains in the protein.

#### Olfactory receptor genes were overrepresented in the PTC dataset

The biological functionality of genes in the two datasets was assessed using Gene Ontology [32] classifications. All three ontologies; biological process, cellular component and molecular function were used for the classification and the FatiGO tool [33] was used to test if any GO classes were overrepresented in the PTC dataset compared to the non-PTC dataset. We found that PTC genes were significantly overrepresented in three GO classes: olfactory receptor activity, sensory perception of smell and G-protein coupled receptor protein signaling pathway (see Table 2 for details). These GO terms are to a large extent overlapping, and 46 of the PTC genes were found in all three GO classes (listed in Additional file 1, Table S2).

**Table 2:** Gene ontology classification of PTC genes.

**Ontology**	**Overrepresented GO term in the PTC dataset**	Number of PTC genes in the GO category*	**Adjusted p-value**
Molecular function	Olfactory receptor activity (GO:0004984)	46	1*10^-6^
Biological process	Sensory perception of smell (GO:0007608)	46	3*10^-7^
Biological process	G-protein coupled receptor protein signaling pathway (GO:0007186)	82	2*10^-5^

* Note that the GO terms overlap. The two groups olfactory receptor activity and sensory perception of smell are identical and these 46 genes are in turn included in the group G-protein coupled receptor activity.

All PTC genes mapping to the GO terms olfactory receptor activity and sensory perception of smell belong to the olfactory receptor (OR) superfamily of genes. There are almost one thousand OR genes known in human, with ~50% of them being pseudogenes [34,35]. The OR genes belong to the G-protein coupled receptor (GPCR) hyperfamily, explaining the presence of G-protein coupled receptor protein signaling pathway in the GO classification. It is generally thought that the number of functional OR genes have been reduced in the primate lineage as compared to other mammals, and different selective mechanisms have been proposed [35-38]. A recent study [39] suggested that humans and chimpanzees have similar numbers of OR genes and also comparable fractions of pseudogenes. The repertoire of OR genes expressed has been suggested to differ between species, allowing for species-specific adaptations of odor perception [39].

Among the 46 PTC genes that were overrepresented in all three GO categories (Table 2 and Table S2), all but two have only a single annotated transcript. This implies that these genes are pseudogenes in the chimpanzee, since all known transcripts have been silenced. For the two genes with several annotated transcripts, the effect may be different. Most likely these genes were not completely switched off but rather the range of available transcripts was altered.

### The effect of PTCs on alternative splicing

A surprisingly high proportion of the genes in the study were found to have PTCs, but not all affected genes represented pseudogenes. Approximately half of the genes in the study have multiple annotated transcripts and the same holds true for the PTC dataset. The majority of these genes have at least one transcript unaffected by the PTC, suggesting that most PTC mutations do not switch off the gene, but rather a specific transcript. Furtermore, the Ensembl prediction that ~50% of the genes have multiple splice variants is likely to be an underestimate since recent studies have shown that nearly all human multiexon genes have multiple splice variants [40,41]. Thus it is reasonable to assume that virtually none of the multiexon genes with PTCs in chimpanzee were entirely silenced by the mutation.

Depending on the location of the PTC, the affected mRNA may either be translated into a truncated protein product or become degraded by NMD [19,20]. The coupling of PTCs and NMD has previously been proposed as a novel mechanism for regulating gene expression [21-24]. By selectively silencing transcripts, the coupled action of PTCs and NMD may modulate gene expression and alter the repertoire of expressed isoforms, thereby providing for species-specific patterns of alternative splicing.

Since there are no *de novo *annotations of chimpanzee genes and transcripts, the present study is based solely on human annotations [42]. It is possible that in some cases where we predict a PTC in a chimpanzee gene, the chimpanzee may instead have different gene structure and/or different splice variants, thereby adding to the transcriptional complexity. To study this in more detail it would be necessary to perform large-scale sequencing of the chimpanzee transcriptome.

### Conclusion

We have performed a genome wide study of premature termination codons (PTCs) in the chimpanzee to estimate the frequency of such events and further characterize the affected genes. We found that ~8% (1,109/13,487) of the genes had at least one transcript affected by a PTC. Indels rather than substitutions were the main cause of PTCs. We observed both inter- and intra-chromosomal fluctuation in the density of PTC genes and this variation was related both to local variations in indel divergence and proximity to the telomere. Within genes, PTCs occurred towards the 5' and 3' ends of the genes, thereby preserving functional domains in the central part of the genes. This indicates that selection against PTCs was stronger in the central and more conserved functional parts of proteins.

Gene Ontology classification revealed that PTC genes were overrepresented in the groups associated with olfaction. This was especially intriguing since the repertoire of olfactory receptors in primates has been extensively studied and it has been shown that many olfactory receptor genes have become pseudo genes in the primate linage [35-37,39]. Premature termination codons are most likely a major factor in the pseudogenisation process.

Approximately half of the genes in this study had multiple annotated transcripts and in most cases the PTC did not affect all of the transcripts. Instead of becoming pseudo genes, the affected genes seemed to have certain transcripts silenced by the PTCs. Transcripts affected by PTCs will either be degraded by NMD or produce truncated protein isoforms. Either way, the PTCs result in a different a transcript repertoire between humans and chimpanzees and PTCs may therefore contribute to the factors determining the phenotypic differences between the species. The phenotypic differences between humans and chimpanzees has been attributed to divergence in protein coding genes [7], gene expression [12-14] and alternative splicing [15] as well as gain and loss of genetic material [8-11]. Indels and substitutions causing PTCs potentially affect several of these mechanisms, in particular the pattern of gene expression and alternative splicing. Moreover, the paired action of PTCs and nonsense-mediated decay has been suggested as a novel mechanism to regulate gene expression [21-24] and the observed frequent occurrence of PTC mutations further support the hypothesis that divergent gene expression and alternative splicing affect the phenotypic divergence.

## Methods

### Selection and filtering of transcripts

Genomic sequence for human (hg18/build 36) and chimpanzee (panTro2) were downloaded from the Ensembl database and annotations of all human protein coding genes and transcripts were likewise obtained from Ensembl (release 50) [42]. Chimpanzee genes and transcripts were inferred from human gene annotations and the coordinates were translated between the genomes using a command line version of liftOver (available from the UCSC Genome Browser [28]). After translation with liftOver, all chimpanzee exons from the same transcript were required to be both on the same chromosome and on the same strand, or else the transcript was removed from further analysis. Exon sequences were concatenated into complete coding sequences for both human and chimpanzee, translated to amino acid sequences and scanned for PTCs in the chimpanzee. To ensure the correctness of the predicted PTCs we applied a number of additional filtering steps (outlined in Figure 1).

(i) All transcripts with Ns in the chimpanzee sequence were removed to exclude regions with low sequence coverage.

(ii) All transcripts with PTCs in human were removed and likewise for transcripts lacking a valid start (M or L) or stop codon in human. The Ensembl set of genes is based on automatic annotation and a minority of transcripts violates the above requirements. This group of transcripts tends to have fewer supporting mRNA/EST sequences and was therefore considered less reliable.

(iii) Transcripts lacking a valid start codon in the chimpanzee were removed since such transcripts were not likely to be coding.

(iv) To account for the varying quality of chimpanzee genome assembly, all transcripts with predicted PTCs were filtered on the sequence quality score. The quality score-track from the UCSC Genome Browser [28] was downloaded and we used a threshold of 40, which corresponds to less then 1 sequencing error per 10,000 bp. The substitution or indel event causing the PTC was determined for all PTC transcripts and we required that a 10 bp region (5 bp upstream and 5 bp downstream) surrounding the event had a quality score > 40. To determine the type of mutational event leading to the PTC, all affected transcripts were first scanned for substitutions. If a nonsense mutation coincided with the predicted PTC, this substitution was regarded as the causative event. Otherwise, the PTC must be caused by an indel event and the first indel in the transcript was inferred to be the cause of the subsequent PTC.

The aim of the filtering process was to make sure that the PTCs were not caused by incorrect gene predictions, lacking sequence or bad quality of the chimpanzee assembly. Furthermore, the filtering removed transcripts with speculative coding capacity. All chimpanzee PTCs that remained after the filtering procedure were considered to be true.

#### Description of the two datasets

The filtering process resulted in two datasets, one group of genes with predicted PTCs in the chimpanzee (the PTC dataset) and one group with no predicted PTCs in the chimpanzee (the non-PTC dataset). The two datasets could be analyzed either at the gene level or at the transcript level and we have chosen the first approach. It is likely that multiple transcripts from a gene will cover almost the same genomic regions and therefore the analyses was done on the gene level to avoid the bias introduced if we sample the same genomic region several times. Approximately half of the genes have more then one annotated transcript and we randomly chose one transcript per gene for the analyses. However, for genes in the PTC-dataset the chosen transcript was required to have a PTC.

### Characterizing genes with PTCs in chimpanzee

#### Annotation of genomic features

To determine if chimpanzee PTC genes share some genomic properties we studied a number of genomic features such as GC-content, repeat content, CpG-islands, segmental duplications, sequence divergence and proximity to the centromere and telomere. Since the studied PTCs occur in the chimpanzee we used annotations based on the chimpanzee genome. GC-content was calculated directly from the chimpanzee genome sequence. Coordinates for CpG-islands and repeats in the chimpanzee genome were downloaded from the UCSC Genome browser (CpG-island track and RepeatMasker track) [28]. Annotations of chimpanzee segmental duplications were obtained from . In this case the segmental duplications had been mapped to an old version of the human genome (hg16, build 34) and liftOver was used to translate the coordinates to the most recent chimpanzee genome. Sequence divergence caused by substitutions and indels was estimated from the pairwise alignment of the human and chimpanzee genomes, also downloaded from UCSC. Substitutions were counted directly from the alignment whereas the indel divergence was estimated in two ways: (i) as the number of indel events or (ii) the number of bp involved in indels (i.e. the number of bp in indels/the number of aligned bp).

#### Regression analysis and choice of window size

In order to investigate regional variation in the density of PTC genes we first estimated the proportion of PTC genes, as well as non-PTC genes, in windows across all chromosomes. To determine the genomic location of genes we used the start coordinates, and genes were counted once in the window where they started. The number of PTC genes, and non-PTC genes, genes in a window was normalized with the total number of genes in the same window. In addition, we analyzed a number of genomic features over the same windows to assess a possible correlation with genes in the two datasets. GC-content, substitutions and indel divergence (calculated as the proportion of bp located in indels) were estimated on the nucleotide levels and thus the occurrence of these features was simply counted within each window. CpG-islands, repeats, segmental duplications and indel events on the other hand span over several nucleotides and in this case the feature was counted once, in the window where the start was located. RepeatMasker [43] classifies repeats into ten different classes and we performed the analyses both with separate repeat classes and with a merged set of all repeats. There were no annotations of the location of chimpanzee centromeres available so the positions were estimated from the pairwise alignment files previously used to determine the sequence divergence. The centromere was inferred to the 1 Mbp-window with zero aligned nucleotides and if several consecutive windows with zero aligned bp occurred the centromere was placed in the middle.

After estimating the proportion of genes in the two datasets, as well as a number of genomic features, we applied a linear regression model. The relationship between each genomic feature and the proportion of genes in the two datasets was evaluated separately. Variables with a higher correlation to PTC genes then to non-PTC genes were kept and included in a multiple linear regression model. All statistical calculations were done in R [44].

Several different widow sizes were evaluated and we settled for 1 Mbp intervals, which has also previously proved to be a reasonable choice for this type of analyses [45]. It may be hypothesized that shorter windows would improve the resolution in the analyses but this was not the case in the present study. Shortening the window length obviously increased the number of windows and resulted in more windows with zero genes in them. We also tried to increase the window size but this did not improve the analysis either. Probably, because larger windows tend to 'average out' the differences between different genomic regions.

#### Location of PTCs within the affected gene and analysis of a possible association with functional protein domains

The exact position of PTCs within the genes was determined and the locations were then normalized with the gene length to be able to compare positions across all genes. The normalized positions of PTCs were merged into relative intervals (0–5%, 5–10% etc of the gene length). Next, the translated sequences of all PTC genes were searched against the Pfam database [31] to examine a possible association between the location of PTCs and the location of functional domains in the protein. The positions of all Pfam matches within a sequence were recorded and the matches were filtered using an e-value of 0.001 as cut off (other cut offs were tried and the results were similar, data not shown). The locations of Pfam matches were calculated in relative intervals (0–5%, 5–10% and so on), similar to the location of PTCs in genes, and matches spanning over several intervals were recorded once in each interval. Finally, we used R [44] to calculate the correlation between PTC positions and functional domains.

#### Gene Ontology classification

The FatiGO tool [33] was used to test for over/under representation of specific GO classes in the PTC dataset, as compared to the non-PTC dataset. All three GO categories [32] (biological process, cellular component and molecular function) were tested.

## Abbreviations

Indel: insertion or deletion of nucleotides in a sequence; Mbp: megabasepairs; NMD: nonsense mediated decay; PTC: premature termination codon.

## Authors' contributions

AW designed the study, performed the analysis and statistics and drafted the paper. UG, LC and TB helped in the design of the study and in writing of the paper. All authors read and approved the final manuscript.

## Supplementary Material

Additional file 1 **Genes affected by PTCs.** The file contains Table S1 which includes the complete PTC gene dataset and Table S2 with the 46 genes found in all three GO categories that were overrepresented in the PTC dataset. Click here for file
